# Cathepsin K: A Novel Diagnostic and Predictive Biomarker for Renal Tumors

**DOI:** 10.3390/cancers13102441

**Published:** 2021-05-18

**Authors:** Anna Caliò, Matteo Brunelli, Stefano Gobbo, Pedram Argani, Enrico Munari, George Netto, Guido Martignoni

**Affiliations:** 1 Department of Diagnostic and Public Health, Section of Pathology, University of Verona, 37134 Verona, Italy; anna.calio@univr.it (A.C.); matteo.brunelli@univr.it (M.B.); 2 Department of Pathology, Pederzoli Hospital, 37019 Peschiera del Garda, Italy; gobo79@gmail.com; 3 Department of Pathology, Johns Hopkins Medical Institutions, Baltimore, MA 21287, USA; pargani@jhmi.edu; 4 Department of Pathology, ASST Spedali Civili of Brescia, 25123 Brescia, Italy; enrico.munari@unibs.it; 5 Department of Pathology, University of Alabama at Birmingham, Birmingham, AL 35233, USA; gnetto@uabmc.edu

**Keywords:** cathepsin K, renal cancers, PEComa, translocation renal cell carcinoma, differential diagnosis, predictive markers, TSC1/TSC2, mTOR pathway, angiomyolipoma

## Abstract

**Simple Summary:**

Our understanding of renal tumors has increased in the last years with the description of several novel entities. The expanding morphological spectrum complicates the pathologist’s diagnosis, often requiring immunohistochemical analysis. The role of cathepsin K immunoexpression is widened as a diagnostic tool in several renal tumors. This review describes the usefulness of cathepsin K in the differential diagnosis of renal neoplasms, highlighting the biological knowledge underpinning its expression. Moreover, cathepsin K seems to be a downstream marker of different genetic alterations, with a possible role as a predictive marker that may prospectively guide the development of therapeutic approaches as a molecular target.

**Abstract:**

Cathepsin K is a papain-like cysteine protease with high matrix-degrading activity. Among several cathepsins, cathepsin K is the most potent mammalian collagenase, mainly expressed by osteoclasts. This review summarizes most of the recent findings of cathepsin K expression, highlighting its role in renal tumors for diagnostic purposes and as a potential molecular target. Indeed, cathepsin K is a recognized diagnostic tool for the identification of TFE3/TFEB-rearranged renal cell carcinoma, TFEB-amplified renal cell carcinoma, and pure epithelioid PEComa/epithelioid angiomyolipoma. More recently, its expression has been observed in a subgroup of eosinophilic renal neoplasms molecularly characterized by *TSC/mTOR* gene mutations. Interestingly, both *TSC* mutations or *TFE3* rearrangement have been reported in pure epithelioid PEComa/epithelioid angiomyolipoma. Therefore, cathepsin K seems to be a downstream marker of TFE3/TFEB rearrangement, TFEB amplification, and mTOR pathway activation. Given the established role of mTOR inhibitors as a pharmacological option in renal cancers, cathepsin K could be of use as a predictive marker of therapy response and as a potential target. In the future, uropathologists may implement the use of cathepsin K to establish a diagnosis among renal tumors with clear cells, papillary architecture, and oncocytic features.

## 1. Introduction

Cathepsin K is an established immunohistochemical marker in the classification of primary renal neoplasms, as demonstrated by the International Society of Urological Pathology (ISUP) recommendations, to identify translocation renal cell carcinoma and the spectrum of perivascular epithelioid lesions, in particular pure epithelioid PEComa/epithelioid angiomyolipoma [1]. However, in the last few years, our evolving understanding of this marker has widened its application potential. In this report, we offer a review of cathepsin K, specifically among renal tumors, highlighting its role in the differential diagnosis and as a possible molecular target.

## 2. Cathepsins

Cathepsins are a group of proteases implicated in a variety of physiological and pathological processes [2]. The name derives from the Greek kathepsein (to digest), which is one of the main functions. Cathepsins are categorized into three different families: serine (cathepsins A and G), aspartate (cathepsins D and E), and cysteine (cathepsins B, C, F, H, K, L, O, S, V, X, and W) proteases. Nowadays, cathepsins are referred to as the latter group and, for the purposes of this review, we will simply call the cysteine lysosomal protease cathepsins. Despite almost all types of cathepsins sharing a common synthetic pathway, they are mainly distinguished by their structures, catalytic mechanisms, and the proteins cleaved [3,4]. To prevent unwanted protein degradation, they are synthesized as inactive precursors (zymogen), which subsequently translocalize from the endoplasmic reticulum through the Golgi apparatus into lysosomes, where they are finally activated [5]. Their activity is regulated by interactions with endogenous inhibitors of the cystatin superfamily, comprising three distinct families, namely cytosolic stefins, extracellular cystatins, and kininogens. Cystatins make cathepsins that are unable to cleave peptide binding to their active site as pseudo-substrates. The equilibrium between free cathepsins and their complexes with inhibitors is critical for the proper functioning of several processes [6]. 

Cathepsins are predominantly localized in the lysosomes, as their optimal activity requires the type of acidic environment seen in the lysosome, but they can also be found in the intracellular and extracellular spaces [2]. It has been demonstrated they are proteolytically active at a higher pH [7], which is important, for instance, for cancer cell invasion. Most cathepsins are ubiquitously expressed in human tissues and are involved in normal cellular protein degradation and turnover, whereas certain cathepsins have a tissue-specific distribution, suggesting more specific functions. For instance, cathepsin K is expressed in osteoclasts, in most epithelial cells, and in synovial fibroblasts in rheumatoid arthritis joints [8]. Cathepsin S is generally expressed in dendritic cells and B cells, and cathepsin W is generally expressed in CD8+ lymphocytes and natural killer cells [9,10]. Considering the kidney, for example, cathepsins B, D, L, and S are the cathepsins mainly involved in the regulation of physiological (glomerular permeability, extracellular matrix homeostasis, autophagy, and apoptosis,) and pathological (glomerulosclerosis and chronic kidney disease acute kidney injury) processes [11]. Although our knowledge is still limited, it has become more and more evident that several human diseases, such as inflammatory and cardiovascular diseases, obesity, neurodegenerative disorders, kidney dysfunction, and cancer, are due to the abnormal activity of cathepsins, indicating their relevance in numerous physiological processes.

## 3. Cathepsin K

Cathepsin K belongs to the papain-like cysteine peptidase family. It is a member of lysosomal cysteine protease, with the main function of mediating bone resorption under normal and pathological conditions. Cathepsin K is encoded by a *CTSK* gene located on chromosome 1q21.3, with eight exons and seven introns, and a similar organization of cathepsin L and cathepsin S. Mutations on the *CTSK* gene lead to pycnodysostosis, an autosomal recessive disease characterized by osteosclerosis with increased bone fragility, dysmorphic facial features, and short stature [12]. From the structural aspect, cathepsin k is a protein composed of 329-aminoacid comprising of an N-terminal signal sequence (15-amino-acid long), a propeptide (99-amino-acid long), and a catalytic unit (215-amino-acid long). The active site of cathepsin K is at the top of the molecule and contains the catalytic dyad cysteine–histidine [13]. 

Cathepsin K degrades collagens at different sites in the N-terminus region, and has recently been named the most potent mammalian collagenolytic endopeptidase [14]. It is mainly secreted by activated osteoclasts to degrade collagen and other matrix proteins during bone resorption, but also in the hematopoietic stem cells mobilization from the endosteal niche [15]. Because of its activity in bone resorption, it has become an important target for the treatment of osteoporosis (bone resorption exceeds bone formation), even though there is no approved drug so far [15,16]. The activity of cathepsin K is modulated by several factors: RANKL, NFAT, and microphthalmia transcription factor (MiTF) enhance cathepsin K expression, and therefore osteoclast formation and bone resorption. On the other hand, interferon (IFN)-gamma, estradiol, calcitonin, and calcium reduce it [17]. 

Beyond the bone, cathepsin K plays a crucial role in the central nervous, respiratory, and cardiovascular systems [18]. It is detected in neurons, as well as in glial cells, in the bronchial and alveolar epithelial cells, and in the alveolar macrophages in the normal lung, and a small amount of cathepsin K is expressed in the normal heart. Its physiological role in cellular protein turnover, collagen degradation, and remodeling of the extracellular matrix may explain the important role of cathepsin K in neurological disorders, cardiac dysfunction, and pulmonary fibrosis [19] (Table 1). It is also expressed in multinucleated giant cells, Langhans cells, and skin fibroblasts, which play an important role in the homeostasis of the dermal extracellular matrix during wound healing [20]. Taken together, the variety of processes in which cathepsin K is involved and the several diseases genetically or epigenetically associated with cathepsin K expression in either mesenchymal or epithelial tissues explain the increasing interest of scientists in this molecule.

In addition, cathepsin K is closely related to cancer, specifically in processes associated with tumor growth, metastasis, and cancer cell invasion, as well as their interactions with the tumor microenvironment [21]. Cathepsin K may degrade extracellular membrane proteins and destroy the elastic lamina of blood vessels, supporting its role in cancer invasion and progression. Moreover, bone matrix degradation by cathepsin K may facilitate the tumor growth of osseous metastasis. Increased expression and activity of cathepsin K have been demonstrated in patients, either with mesenchymal tumors or epithelial neoplasms, such as bone cancer, prostate cancer [22], breast cancer [23], melanoma [24], colorectal cancer [25], and lung cancer [26]. The relationship between cathepsin K over-expression in the tumor microenvironment and tumor progression has been reported, and it is widely accepted that cathepsin K over-expression is associated with cancer metastatic disease with a potential prognostic value [27]. Diagnostically, the utility of the immunoexpression of cathepsin K has been limited to large series (overall 726) of adenocarcinomas of various organs, reported as single cases with focal labeling in the adrenal cortical carcinomas, urothelial carcinomas, esophageal adenocarcinomas, ovarian serous carcinomas, and pancreatic adenocarcinomas [28]. Although negative in the neoplastic cell, staining for cathepsin K is not uncommonly seen in the peritumoral stroma of adenocarcinomas arising in different organs (esophagus, stomach, colon, pancreas, biliary tract, lung, breast, and ovary), especially in the elongated spindle cells present in the desmoplastic stroma, morphologically considered as reactive myofibroblasts. Conversely to epithelial neoplasms in which cathepsin K has an important role in the differential diagnosis, in the mesenchymal tumor its immuno-expression is not discriminatory. Positive reactions can be found in the alveolar soft part sarcoma, histiocytic lesions (juvenile xanthogranuloma and Langerhans cell histiocytosis), giant cell tumors, granular cell tumors, and gastrointestinal stromal tumors, as well as numerous additional sarcomas including Kaposi sarcoma, liposarcoma, chondrosarcoma, undifferentiated pleomorphic sarcoma, and leiomyosarcoma [28].

## 4. Cathepsin K and Renal Tumors

Although the role of cathepsin K is limited in the physiological processes of the kidney, it has been broadly studied in renal tumors since its first description in translocation renal cell carcinoma in 2009 [29]. Cathepsin K immunolabelling is not observed in the most common adult renal cell carcinomas (clear cell renal cell carcinoma, papillary renal cell carcinoma, and chromophobe renal cell carcinoma), but it is commonly detected in a subset of renal tumors, as illustrated below (Table 2).

### 4.1. Cathepsin K and Translocation Renal Cell Carcinoma

**Figure 1:**
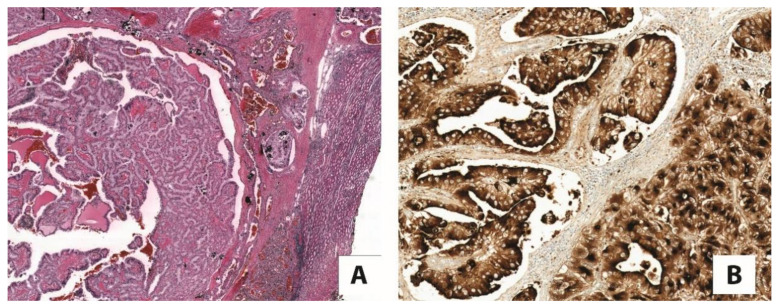
An example of (**A**) TFE3-rearranged renal cell carcinoma with papillary architecture (5×), (**B**) strongly and diffusely positive for cathepsin K (20×).

Translocation renal cell carcinomas comprise two different tumors molecularly characterized by specific gene translocations, namely TFE3-rearranged renal cell carcinoma, the most common subtype, and TFEB-rearranged renal cell carcinoma. As evidenced by their nomenclatures, they harbor *TFE3* and *TFEB* gene fusions, respectively [28]. Initially, they were recognized in childhood; however, these neoplasms can arise in adults as well. TFE3-rearranged renal cell carcinomas display heterogeneous morphological features simulating most of the subtypes of renal cell carcinoma. The most distinctive histological characteristic of TFEB-rearranged renal cell carcinoma is the biphasic appearance: nests of large epithelioid cells and smaller cells clustered around hyaline nodules formed by a basement membrane material that is collagen IV positive. Nevertheless, it is widely accepted that both TFE3-rearranged renal cell carcinoma and TFEB-rearranged renal cell carcinoma may show a broad range of morphology, resulting in a challenging differential diagnosis. Currently, cathepsin K immunostaining is generally used by uropathologists to reach the proper diagnosis of translocation renal cell carcinoma [1,29]. Indeed, cathepsin K is expressed in roughly half of TFE3-rearranged renal cell carcinomas, and is observed in virtually all TFEB-rearranged renal cell carcinomas (Figure 1). In the beginning, the idea of the cathepsin K expression in translocation renal cell carcinoma was postulated based on the consistent ability of MiTF to modulate the cathepsin K gene promoter in osteoclasts. As the target DNA sequences of MiTF overlap with those of TFE3 and TFEB, it has been hypothesized that the overexpressed TFE3 fusion proteins or native TFEB in these renal cell carcinomas may have the same effect on the promoter of cathepsin K. 

Over the last years, several partner genes involved in translocation with the *TFE3* gene, and more recently with *TFEB* gene, have been reported. In TFEB-rearranged renal cell carcinoma, cathepsin K staining is confirmed to be reliable, with strong and diffuse immunolabelling in the neoplastic cells, regardless of the fusion partner gene, such as *MALAT1*, *ACTB,* and *NEAT1* [30]. Conversely, cathepsin K is unevenly observed in TFE3-rearranged renal cell carcinomas. Among the three most common fusions detected in TFE3-rearranged renal cell carcinoma, cathepsin K has been shown to be generally positive in *PRCC-TFE3* rearranged renal cell carcinomas, variably expressed in *SFPQ-TFE3* rearranged renal cell carcinomas, and negative in *ASPCSR1-TFE3* rearranged renal cell carcinomas. Interestingly, *ASPCSR1-TFE3* gene fusion is also characteristic of alveolar soft part sarcoma, a rare soft tissue sarcoma, which is strongly positive for cathepsin K [31]. The different expression of cathepsin K between alveolar soft part sarcoma and *ASPSCR1-TFE3* rearranged renal cell carcinoma has been explained by a subtle difference in the chromosome translocation, unbalanced in alveolar soft part sarcoma, and consistently balanced in *ASPSCR1-TFE3* rearranged renal cell carcinoma. To further complicate matters, in the last years, cases of *PRCC-TFE3* rearranged renal cell carcinoma negative for cathepsin K and cases of *ASPCSR1-TFE3* rearranged renal cell carcinoma positive for cathepsin K have been reported [31,32]. Interestingly, the *PRCC* gene is located on chromosome 1q23.1 close to cathepsin K gene, located on chromosome 1q21.3, as mentioned above. The cathepsin K overexpression may be the result of a disruption of the cathepsin K gene due to chromosome translocation and independent of the function of the *PRCC-TFE3* fusion protein. However, this explanation seems unlikely, because cathepsin K is ∼6 MB away from the breaking point in the *PRCC-TFE3* rearranged renal cell carcinoma and cases negative for cathepsin K are recorded. Among the novel fusions, none of the *NONO-TFE3* rearranged renal cell carcinomas have been shown to express cathepsin K, whereas *RBM10-TFE3* rearranged renal cell carcinoma [33] and some *MED15-TFE3* [34] rearranged renal cell carcinoma are reactive for this marker. Overall, these findings would suggest that over-expressed native TFEB consistently activates cathepsin K expression like MITF does, but that only some TFE3 fusion proteins do.

### 4.2. Cathepsin K and TFEB-Amplified Renal Cell Carcinoma

**Figure 2:**
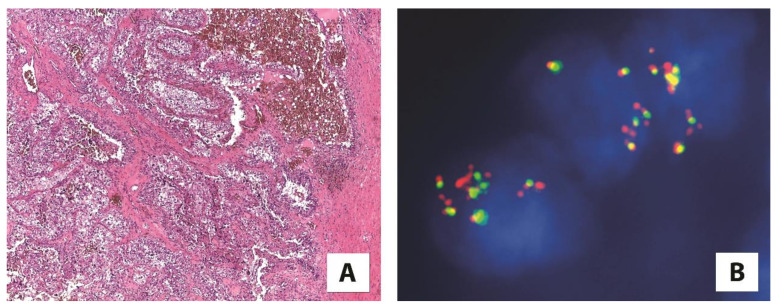
(**A**) A high-grade renal cell carcinoma showing (5×) (**B**) *TFEB* amplification by fluorescence in situ hybridization (FISH) (100×).

Besides renal cell carcinoma with *TFEB* translocation, TFEB-amplified renal cell carcinoma is a distinct group of tumors with amplification of the 6p21 locus harboring *TFEB.* The *TFEB* amplification (commonly observed by fluorescence in situ hybridization) leads, as downstream effects, to TFEB overexpression (Figure 2) [35,36,37,38,39]. These tumors usually occur in older patients compared with TFEB-rearranged renal cell carcinoma, and frequently demonstrate a high-grade/poorly differentiated morphology, often with oncocytic features, and aggressive behavior as an advanced local stage or metastatic disease [37,39,40,41]. The occurrence of *TFEB* amplification in renal cell carcinoma seems to not be related to *TFEB* rearrangement. Nevertheless, few cases of TFEB-rearranged renal cell carcinomas with concomitant *TFEB* amplification have been described [35,42]. TFEB expression in tumors with *TFEB* amplification is lower compared with TFEB-rearranged renal cell carcinomas. Moreover, among the latter, when the classic biphasic morphology was observed, a higher TFEB expression was reported [40]. However, for *TFEB* gene translocation, *TFEB* amplification results in TFEB hyperexpression and subsequent immunohistochemical labeling of cathepsin K [41].

### 4.3. Cathepsin K and Tumors with an Altered mTOR Pathway

Recently, comprehensive genomic analyses have described the relative frequency of tuberous sclerosis complex 1 and 2 *(TSC1/TSC2)* gene mutations, drawing attention to the role of the mammalian target of the rapamycin (mTOR) pathway in different renal tumors. Interestingly, cathepsin K is closely related to the mTOR signaling pathway. Recent studies have demonstrated that mTOR inhibitors may control the functional activities of osteoclasts, such as the expression of cathepsin K [43], and the inhibition of cathepsin K can considerably decrease the phosphorylation of mTOR at S2448 in Caki cells [44]. Hence, the title of this subheading, Cathepsin K and Tumors with an Altered mTOR Pathway, includes angiomyolipoma and pure epithelioid PEComa/epithelioid angiomyolipoma, eosinophilic solid and cystic renal cell carcinoma, and high-grade oncocytic tumor/sporadic renal cell carcinomas with eosinophilic and vacuolated cells/eosinophilic vacuolated tumors, which are all characterized by a dysregulation of the mTOR pathway and cathepsin K expression.

### 4.4. Cathepsin K and PEC Tumors

The perivascular epithelioid cell (PEC) is thought to be the cell of origin of a group of tumors called PEComas, which, in the kidney, principally includes angiomyolipoma, intraglomerular lesions, angiomyolipoma with epithelial cysts, and pure epithelioid PEComa/epithelioid angiomyolipoma [45,46]. 

**Figure 3:**
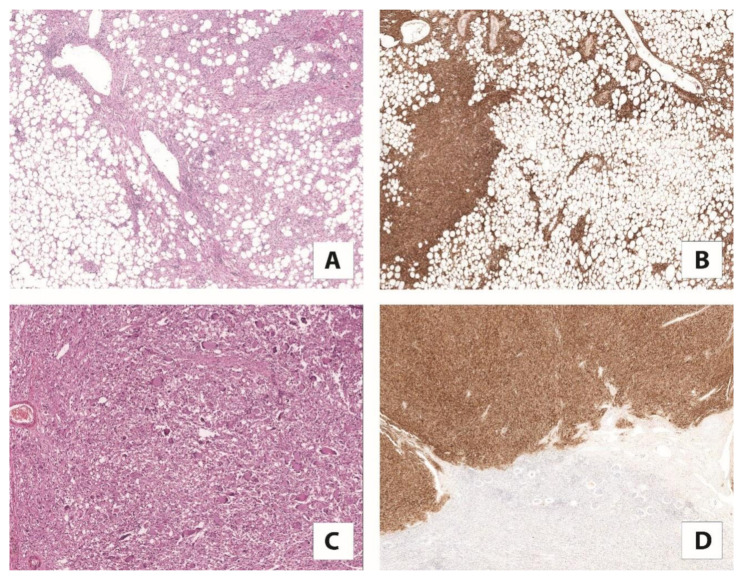
(**A**) Classic angiomyolipoma composed of vessels, smooth muscle, and adipose tissue with (5×) (**B**) a strong expression of cathepsin K (5×). (**C**) Pure epithelioid PEComa/epithelioid angiomyolipoma mainly consists of epithelioid cells (10×) (**D**) diffusely positive for cathepsin K (5×).

Angiomyolipoma is the most common renal mesenchymal tumor characterized by a variable proportion of adipose tissue, spindle and epithelioid smooth muscle cells, and thick-walled blood vessels. Initially considered a hamartoma rather than a true neoplasm, nowadays, it is known that the primary driver genetic event for its development is the biallelic inactivation of *TSC2,* or, less frequently, the *TSC1* gene, demonstrated in up to 94% of cases [47]. Increasing evidence supports the idea that angiomyolipoma is the prototype of lesions in which the mTOR pathway is hyperactivated due to *TSC1/TSC2* gene alterations [45,47]. Of note, the expression of cathepsin K in osteoclasts is regulated by MiTF, a transcription factor expressed in angiomyolipoma, which is significantly reduced by mTOR inhibitors [48]. Based on these observations, it was expected that cathepsin K would be expressed in the angiomyolipoma (Figure 3) [49]. This hypothesis was correct and, to date, it has been widely demonstrated. Cathepsin K is constantly and strongly expressed in all the renal PEComas and it is considered a favorite marker for their identification, especially to confirm diagnosis on needle biopsies [32,49].

**Figure 4:**
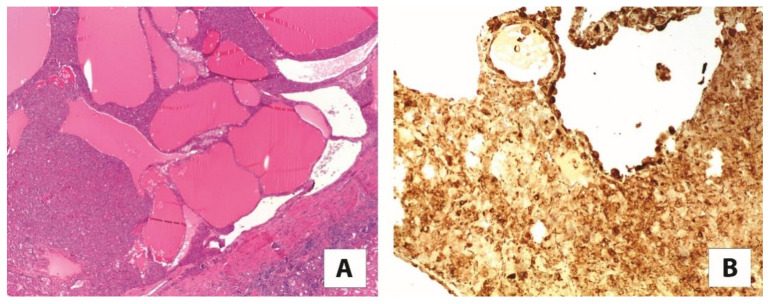
(**A**) The most common morphological features of eosinophilic solid and cystic renal cell carcinoma (2×). (**B**) The neoplastic cells are reactive for cathepsin K (10×).

Pure epithelioid PEComa/epithelioid angiomyolipoma is a rare variant of angiomyolipoma, which mainly consists of epithelioid cells, and can pursue an aggressive behavior. Like classic angiomyolipoma, mutations in the *TSC2* gene have been reported in pure epithelioid PEComa/epithelioid angiomyolipoma. Additionally, *TFE3* gene fusion has been described, as in TFE3-rearranged renal cell carcinoma [50]. Pure epithelioid PEComa/epithelioid angiomyolipoma harboring *TFE3* gene translocation has mostly *SFPQ* as the partner gene. Conversely to TFE3-rearranged renal cell carcinoma, pure epithelioid PEComa/epithelioid angiomyolipoma constantly express cathepsin K (Figure 4), regardless of the type of translocation. One rather obvious explanation is the differences in the cell of origin, which justify the differential expression of cathepsin K when the same translocation occurs.

Ultimately, intraglomerular lesions are a morphological finding that strongly suggests the presence of tuberous sclerosis or TSC2/PDK1 contiguous gene syndrome [51]. They consist of minute nodules made up of few adipocytes and epithelioid smooth muscle cells within the slightly compressed glomerular capillary tuft, and are identified with hematoxylin and eosin. Those lesions are negative for melanogenesis markers (HMB45 and Melan-A), but they have been demonstrated to be positive for cathepsin K [49] and, more recently, for STING [52]. Therefore, cathepsin K can be valuable to detect them, highlighting the presence of microscopic lesions, which indicate the possibility of a diagnosis of tuberous sclerosis.

### 4.5. Cathepsin K and Eosinophilic Solid and Cystic Renal Cell Carcinoma

Eosinophilic solid and cystic renal cell carcinoma is a recently characterized entity that exhibits well-defined clinical, pathological, immunohistochemical, and molecular features [53,54]. It typically occurs in females as sporadic tumors and generally exhibits indolent behavior, although metastatic cases can occur, which imply patient surveillance. A hereditary form arising in patients with tuberous sclerosis complex has been documented. As the name indicates, the tumor is made up of voluminous eosinophilic cells with granular cytoplasmic “stippling” with round nuclei and not prominent nucleoli arranged in a solid and cystic architecture. Foamy macrophages, as admixed or in small clusters, are usually encountered. The somatic bi-allelic loss or mutually exclusive mutation of *TSC1*/*TSC2* genes is the constant molecular alteration of such tumors [55,56], and has even been evaluated to be completely embedded in multiple samples of tumors [57]. Using immunohistochemistry, besides the cytokeratin 20 positivity/cytokeratin 7 negativity, staining for cathepsin K is reported, in either a diffuse or focal pattern (Figure 4) [58,59]. Again, *TSC1*/*TSC2* gene mutation leading to the activation of mTOR pathway may explain cathepsin K immunohistochemical expression.

### 4.6. Cathepsin K and High-Grade Oncocytic Tumor/Sporadic Renal Cell Carcinomas with Eosinophilic and Vacuolated Cells/Eosinophilic Vacuolated Tumor

**Figure 5:**
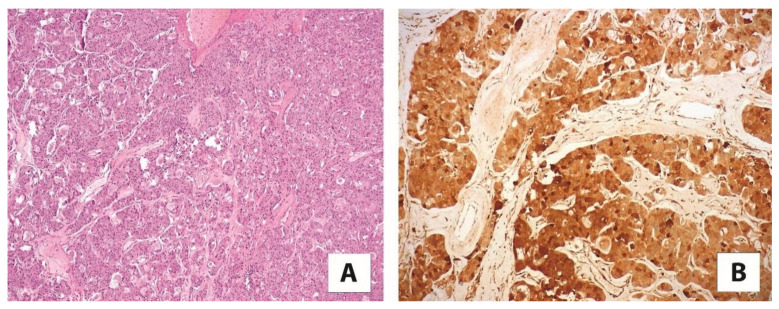
(**A**) A high-grade oncocytic tumor/sporadic renal cell carcinoma with eosinophilic and vacuolated cells (5×) (**B**) immunolabelling for cathepsin K (20×).

A separate subtype of renal tumors characterized by eosinophilic cells and mutations in the TSC/mTOR pathway is emerging. In 2019, Chen et al. described seven cases designated as “sporadic renal cell carcinomas with eosinophilic and vacuolated cells”, characterized by cathepsin K immunoexpression and inactivating mutations of the *TSC2* and *mTOR* activating mutations [60]. The year prior, He and colleagues reported a series of 14 cases termed “high-grade oncocytic tumor (HOT)”, which shared the morphologic features with the tumors described above and stained for cathepsin K, although a mutational analysis was not performed [61]. Those tumors are conceivably viewed as the same entity characterized by defined morphology, immunolabelling for cathepsin K (Figure 5), and *TSC*/*mTOR* gene alteration. Of note, a few months ago, the Genitourinary Pathology Society (GUPS) proposed the name “eosinophilic vacuolated tumor” (EVT) for this distinct entity [62]. Although primarily sporadic, one such tumor has been described in tuberous sclerosis [63]. So far, all cases in the relatively small series described have demonstrated indolent behavior. We recently came across a few aggressive cases (personal communication).

Recently, it has been proposed to create a category of “oncocytic/chromophobe renal cell carcinoma, NOS” for all eosinophilic renal tumors that do not fit into the defined entities of oncocytoma and chromophobe renal cell carcinoma [64]. Within this category, additional molecular studies should be performed for the potential identification of *TSC1/TSC2* and *mTOR* gene mutations. In this scenario, cathepsin K immunoreactivity could further select the eosinophilic renal cell tumors harboring such molecular alterations.

## 5. Cathepsin K in the Differential Diagnosis

Cathepsin K is a valuable marker in the differential diagnosis of renal cell carcinoma (Table 3). Its usefulness has been widely recognized, being included, together with a handful of immunohistochemical markers, in best practice recommendations in immunohistochemical panels to classify primary renal neoplasm [1,27]. 

Among the renal cell carcinomas with clear cells and a papillary architecture, cathepsin K is the most reliable immunohistochemical tool to discern translocation renal cell carcinoma from the most common clear cell renal cell carcinomas and papillary renal cell carcinomas, which are consistently negative for this marker [41,65]. Meaningfully, as above mentioned, immunolabeling for cathepsin K is observed in approximately half of TFE3-rearranged renal cell carcinomas, the neoplasm most confused with clear cell renal cell carcinomas and papillary renal cell carcinoma [66]. Thanks to the consistent reactivity of neoplastic cells for cathepsin K in TFEB-rearranged renal cell carcinoma, it is easily distinguishable from the usual types of renal cell carcinomas [67]. Differentiating pure epithelioid PEComa/epithelioid angiomyolipoma from TFEB-rearranged renal cell carcinoma is challenging, as the two entities share the immunohistochemical expression of cathepsin K and melanocytic markers, such as HMB45 and Melan-A. PAX8 immunostaining and CD68 (PG-M1) negativity support the diagnosis of TFEB-rearranged renal cell carcinoma, whereas pure epithelioid PEComa/epithelioid angiomyolipoma has the opposite immunoreactivity, being negative for PAX8 and positive for CD68 (PG-M1) [68].

Among the renal tumors characterized by eosinophilic cells, cathepsin K is observed in eosinophilic solid and cystic renal cell carcinoma and high-grade oncocytic tumor/sporadic renal cell carcinomas with eosinophilic and vacuolated cells/eosinophilic vacuolated tumors, along with translocation renal cell carcinoma, which may mimic almost all subtypes of renal cell carcinoma because of the broad range of morphologies. On the other hand, the most common eosinophilic renal tumors (oncocytoma, eosinophilic variant of chromophobe renal cell carcinoma, and oncocytic papillary renal cell carcinoma) are considered negative for cathepsin K. However, a recent study describes the immunohistochemical expression of cathepsin K in a small series of oncocytoma (13 cases) and chromophobe renal cell carcinoma (13 cases) [69]. As the authors stated, the differences to the prior results may be explained by the use of a different clone (clone EPR19992). Although clone EPR19992 and clone 3F9 (the most used) were produced by the same manufacturer, the sequence and location of the epitope in this newer antibody are unknown. In our hands, oncocytoma and chromophobe renal cell carcinoma are negative for cathepsin K (clone 3F9). Focal staining (less than 5% of neoplastic cells) is observed in one of fifty-four oncocytomas and three of fifty-six chromophobe renal cell carcinomas. Overall, cathepsin K is helpful for recognizing these types of tumor entities and distinguishing them from other eosinophilic renal tumors most frequently encountered in daily clinical practice.

Finally, it is worth noting the presence of cathepsin K labeling in the capillaries and the associated macrophages in most renal cell neoplasms, as an internal control to evaluate the quality of the staining.

## 6. Conclusions

**Figure 6:**
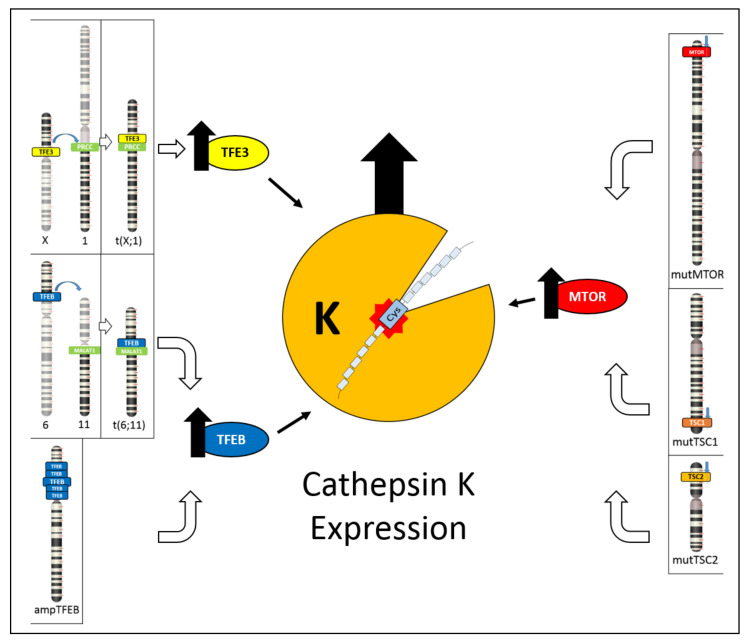
Schematic illustration showing the different mechanisms leading to cathepsin K expression. On the left, TFE3 hyperexpression due to *TFE3* gene translocation and TFEB hyperexpression due to either *TFEB* gene translocation or *TFEB* gene amplification cause cathepsin K expression. On the right, inactivating mutations of *TSC1/TSC2* genes or activating mutations of *mTOR* gene causes mTOR pathway activation, resulting in cathepsin K expression.

By exploring the novel insights into the cathepsin K expression, this review has the aim to highlight its usefulness in the differential diagnosis of renal tumors and to underline the relationship between cathepsin K and TFE3 translocations, TFEB translocation/amplification, and the cathepsin K and mTOR pathway (Figure 6). TFE3 hyperexpression resulting from *TFE3* gene translocation and the overexpression of TFEB due to either *TFEB* gene translocation or *TFEB* gene amplification cause cathepsin K expression [49]. On the other hand, inactivating mutations of *TSC1/TSC2* genes or activating mutations of the *mTOR* gene cause mTOR pathway activation, possibly resulting in cathepsin K expression [70,71,72]. 

This aspect would be also interesting from a therapeutic point of view, especially in this era of personalized medicine, in which identifying biomarkers that can better predict response to therapy is crucial. mTOR inhibitors are an important pharmacological option for patients with tumors bearing TSC1/TSC2 mutations, and cathepsin K can be useful to detect these tumors. Understanding the precise interactions between cathepsin K and the mTOR pathway remains unknown and further studies are warranted to shed light on such mechanisms.

## Figures and Tables

**Table 1:** Cathepsin K in human diseases (except kidney).

Organ/System	Non-Neoplastic	Neoplastic
Respiratory tract	lung fibrosis	lung carcinoma(stromal cells)
Bone	pycnodysostosis	giant cell tumors
	osteoporosis	chondrosarcoma
	rheumatoid artritis	
Central nervous system	stroke	
	schizophrenia	
	cerebral aneurism	
Cardiovascular system	cardiac dysfunction	
	myocardial infarction	
	atherosclerosis	
Skin	wound healing	melanoma
Other	autoimmune diseases	alveolar soft part sarcoma
	overweight/obesity	histiocytic lesions
		GIST
		sarcomas

**Table 2:** Renal tumors with expression of cathepsin K.

Histotype	Morphological Features	Molecular Alteration	Cathepsin K	HMB45/Melan-A	PAX8	CD68(PG-M1)
TFE3-rearranged RCC	clear cells in nests	*ASPCR1-TFE3* fusion	negative	variable	positive	negative
	papillary architecture	*PRCC-TFE3* fusion	positive	variable	positive	negative
	variable	*SFPQ-TFE3* fusion	variable	variable	positive	negative
TFEB-rearranged RCC	biphasic appearance	*MALAT1-TFEB* fusion	positive	positive	positive	negative
TFEB-amplified RCC	high grade	*TFEB* amplification	positive	positive	positive	negative
PEComa	epithelioid cells	*TSC2* mutation	positive	positive	negative	positive
	epithelioid cells	*SFPQ-TFE3* fusion	positive	positive	negative	positive
ESC-RCC	eosinophilic solid and cystic	*TSC1/TSC2* mutation	positive	negative	positive	n.a.
EVT	high grade oncocytic	*TSC2/mTOR* mutation	positive	negative	positive	n.a.

RCC: renal cell carcinoma; ESC: eosinophilic solid and cystic; EVT: eosinophilic vacuolated tumor; n.a.: not available.

**Table 3:** Cathepsin k in differential diagnosis among renal tumors.

Pattern	Histotype	Cathepsin K
clear cell	clear cell RCC	negative
	clear cell papillary RCC	negative
	chromophobe RCC	negative
	TFE3-rearranged RCC	variable
	TFEB-rearranged RCC	positive
	PEComa	positive
papillary architecture	papillary RCC	negative
	clear cell papillary RCC	negative
	TFE3-rearranged RCC	variable
	TFEB-rearranged RCC	positive
oncocytic cells	oncocytoma	negative
	chromophobe RCC	negative
	TFE3-rearranged RCC	variable
	TFEB-rearranged RCC	positive
	PEComa	positive
	ESC-RCC	positive
	eosinophilic vacuolated tumor	positive

RCC—renal cell carcinoma; ESC—eosinophilic solid and cystic.

## References

[1] Reuter V.E., Argani P., Zhou M., Delahunt B. (2014). Members of the IIiDUPG. Best practices recommendations in the application of immunohistochemistry in the kidney tumors: Report from the International Society of Urologic Pathology consensus conference. Am. J. Surg. Pathol..

[2] Turk V., Stoka V., Vasiljeva O., Renko M., Sun T., Turk B., Turk D. (2012). Cysteine cathepsins: From structure, function and regulation to new frontiers. Biochim. Biophys. Acta.

[3] Ishidoh K., Kominami E. (2002). Processing and activation of lysosomal proteinases. Biol. Chem..

[4] Wiederanders B., Kaulmann G., Schilling K. (2003). Functions of propeptide parts in cysteine proteases. Curr. Protein Pept. Sci..

[5] Shamsi A., Bano B. (2017). Journey of cystatins from being mere thiol protease inhibitors to at heart of many pathological conditions. Int. J. Biol. Macromol..

[6] Mohamed M.M., Sloane B.F. (2006). Cysteine cathepsins: Multifunctional enzymes in cancer. Nat. Rev. Cancer..

[7] Salminen-Mankonen H.J., Morko J., Vuorio E. (2007). Role of cathepsin K in normal joints and in the development of arthritis. Curr. Drug Targets.

[8] Wex T., Wex H., Hartig R., Wilhelmsen S., Malfertheiner P. (2003). Functional involvement of cathepsin W in the cytotoxic activity of NK-92 cells. FEBS Lett..

[9] Hsing L.C., Rudensky A.Y. (2005). The lysosomal cysteine proteases in MHC class II antigen presentation. Immunol. Rev..

[10] Cocchiaro P., de Pasquale V., della Morte R., Tafuri S., Avallone L., Pizard A., Moles A., Pavone L.M. (2017). The multifaceted role of the lysosomal protease cathepsins in kidney disease. Front. Cell. Dev. Biol..

[11] Motyckova G., Fisher D.E. (2002). Pycnodysostosis: Role and regulation of cathepsin K in osteoclast function and human disease. Curr. Mol. Med..

[12] McGrath M.E., Klaus J.L., Barnes M.G., Bromme D. (1997). Crystal structure of human cathepsin K complexed with a potent inhibitor. Nat. Struct. Biol..

[13] Novinec M., Lenarcic B. (2013). Cathepsin K: A unique collagenolytic cysteine peptidase. Biol. Chem..

[14] Costa A.G., Cusano N.E., Silva B.C., Cremers S., Bilezikian J.P. (2011). Cathepsin K: Its skeletal actions and role as a therapeutic target in osteoporosis. Nat. Rev. Rheumatol..

[15] Garber K. (2016). Two pioneering osteoporosis drugs finally approach approval. Nat. Rev. Drug Discov..

[16] Troen B.R. (2006). The regulation of cathepsin K gene expression. Ann. N. Y. Acad. Sci..

[17] Dai R., Wu Z., Chu H.Y., Lu J., Lyu A., Liu J., Zhang G. (2020). Cathepsin K: The action in and beyond bone. Front. Cell. Dev. Biol..

[18] Buhling F., Rocken C., Brasch F., Hartig R., Yasuda Y., Saftig P., Bromme D., Welte T. (2004). Pivotal role of cathepsin K in lung fibrosis. Am. J. Pathol..

[19] Runger T.M., Quintanilla-Dieck M.J., Bhawan J. (2007). Role of cathepsin K in the turnover of the dermal extracellular matrix during scar formation. J. Investig. Dermatol..

[20] Verbovsek U., van Noorden C.J., Lah T.T. (2015). Complexity of cancer protease biology: Cathepsin K expression and function in cancer progression. Semin. Cancer Biol..

[21] Brubaker K.D., Vessella R.L., True L.D., Thomas R., Corey E. (2003). Cathepsin K mRNA and protein expression in prostate cancer progression. J. Bone Miner. Res..

[22] Littlewood-Evans A.J., Bilbe G., Bowler W.B., Farley D., Wlodarski B., Kokubo T., Inaoka T., Sloane J., Evans D.B., Gallagher J.A. (1997). The osteoclast-associated protease cathepsin K is expressed in human breast carcinoma. Cancer Res..

[23] Petricevic S.J., Pavlovic A., Capkun V., Becic K., Durdov M.G. (2017). Cathepsin K expression in melanoma is associated with metastases. Histol. Histopathol..

[24] Li R., Zhou R., Wang H., Wang H., Li W., Pan M., Yao X., Zhan W., Yang S., Xu L. (2019). Gut microbiota-stimulated cathepsin K secretion mediates TLR4-dependent M2 macrophage polarization and promotes tumor metastasis in colorectal cancer. Cell Death Differ..

[25] Yang H., Heyer J., Zhao H., Liang S., Guo R., Zhong L. (2020). The potential role of Cathepsin K in non-small cell lung cancer. Molecules.

[26] Joyce J.A., Baruch A., Chehade K., Meyer-Morse N., Giraudo E., Tsai F.Y., Greenbaum D.C., Hager J.H., Bogyo M., Hanahan D. (2004). Cathepsin cysteine proteases are effectors of invasive growth and angiogenesis during multistage tumorigenesis. Cancer Cell..

[27] Zheng G., Martignoni G., Antonescu C., Montgomery E., Eberhart C., Netto G., Taube J., Westra W., Epstein J.I., Lotan T. (2013). A broad survey of cathepsin K immunoreactivity in human neoplasms. Am. J. Clin. Pathol..

[28] Argani P. (2015). MiT family translocation renal cell carcinoma. Semin. Diagn. Pathol..

[29] Akgul M., Williamson S.R., Ertoy D., Argani P., Gupta S., Calio A., Reuter V., Tickoo S., Al-Ahmadie H.A., Netto G.J. (2021). Diagnostic approach in TFE3-rearranged renal cell carcinoma: A multi-institutional international survey. J. Clin. Pathol..

[30] Calio A., Harada S., Brunelli M., Pedron S., Segala D., Portillo S.C., Magi-Galluzzi C., Netto G.J., Mackinnon A.C., Martignoni G. (2020). TFEB rearranged renal cell carcinoma. A clinicopathologic and molecular study of 13 cases. Tumors harboring MALAT1-TFE, B.; ACTB-TFE, B.; and the novel NEAT1-TFEB translocations constantly express PDL1. Mod. Pathol..

[31] Martignoni G., Gobbo S., Camparo P., Brunelli M., Munari E., Segala D., Pea M., Bonetti F., Illei P.B., Netto G.J. (2011). Differential expression of cathepsin K in neoplasms harboring TFE3 gene fusions. Mod. Pathol..

[32] Argani P., Zhong M., Reuter V.E., Fallon J.T., Epstein J.I., Netto G.J., Antonescu C.R. (2016). TFE3-Fusion Variant Analysis Defines Specific Clinicopathologic Associations Among Xp11 Translocation Cancers. Am. J. Surg. Pathol..

[33] Argani P., Zhang L., Reuter V.E., Tickoo S.K., Antonescu C.R. (2017). RBM10-TFE3 renal cell carcinoma: A potential diagnostic pitfall due to cryptic intrachromosomal Xp11.2 inversion resulting in false-negative TFE3 FISH. Am. J. Surg. Pathol..

[34] Song Y., Yin X., Xia Q., Zheng L., Yao J., Zeng H., Nie L., Gong J., Zhou Q., Chen N. (2021). Xp11 translocation renal cell carcinoma with morphological features mimicking multilocular cystic renal neoplasm of low malignant potential: A series of six cases with molecular analysis. J. Clin. Pathol..

[35] Argani P., Reuter V.E., Zhang L., Sung Y.S., Ning Y., Epstein J.I., Netto J.G., Antonescu C.R. (2016). TFEB-amplified renal cell carcinomas: An Aggressive molecular subset demonstrating variable melanocytic marker expression and morphologic heterogeneity. Am. J. Surg. Pathol..

[36] Williamson S.R., Grignon D.J., Cheng L., Favazza L., Gondim D.D., Carskadon S., Gupta N.S., Chitale D.A., Kalyana-Sundaram S., Palanisamy N. (2017). Renal cell carcinoma with chromosome 6p amplification including the TFEB gene: A novel mechanism of tumor pathogenesis?. Am. J. Surg. Pathol..

[37] Gupta S., Johnson S.H., Vasmatzis G., Porath B., Rustin J.G., Rao P., Costello B.A., Leibovich B.C., Thompson R.H., Cheville J.C. (2017). TFEB-VEGFA (6p21.1) co-amplified renal cell carcinoma: A distinct entity with potential implications for clinical management. Mod. Pathol..

[38] Skala S.L., Xiao H., Udager A.M., Dhanasekaran S.M., Shukla S., Zhang Y., Landau C., Shao L., Roulston D., Wang L. (2017). Detection of 6 TFEB-amplified renal cell carcinomas and 25 renal cell carcinomas with MITF translocations: Systematic morphologic analysis of 85 cases evaluated by clinical TFE3 and TFEB FISH assays. Mod. Pathol..

[39] Mendel L., Ambrosetti D., Bodokh Y., Ngo-Mai M., Durand M., Simbsler-Michel C., Delhorbe M., Amiel J., Pedeutour F. (2018). Comprehensive study of three novel cases of TFEB -amplified renal cell carcinoma and review of the literature: Evidence for a specific entity with poor outcome. Genes Chromosom. Cancer.

[40] Caliò A., Brunelli M., Segala D., Pedron S., Doglioni C., Argani P., Martignoni G. (2018). VEGFA amplification/increased gene copy number and VEGFA mRNA expression in renal cell carcinoma with TFEB gene alterations. Mod. Pathol..

[41] Gupta S., Argani P., Jungbluth A.A., Chen Y.B., Tickoo S.K., Fine S.W., Gopalan A., Al-Ahmadie H.A., Sirintrapun S.J., Sanchez A. (2019). TFEB expression profiling in renal cell carcinomas: Clinicopathologic correlations. Am. J. Surg. Pathol..

[42] Peckova K., Vanecek T., Martinek P., Spagnolo D., Kuroda N., Brunelli M., Vranic S., Djuricic S., Rotterova P., Daum O. (2014). Aggressive and nonaggressive translocation t(6;11) renal cell carcinoma: Comparative study of 6 cases and review of the literature. Ann. Diagn. Pathol..

[43] Dai Q., Xie F., Han Y., Ma X., Zhou S., Jiang L., Zou W., Wang J. (2017). Inactivation of Regulatory-associated Protein of mTOR (Raptor)/Mammalian Target of Rapamycin Complex 1 (mTORC1) Signaling in Osteoclasts Increases Bone Mass by Inhibiting Osteoclast Differentiation in Mice. J. Biol. Chem..

[44] Seo S.U., Woo S.M., Kim M.W., Lee H.-S., Kim S.H., Kang S.C., Lee E.-W., Min K.-J., Kwon T.K. (2020). Cathepsin K inhibition-induced mitochondrial ROS enhances sensitivity of cancer cells to anti-cancer drugs through USP27x-mediated Bim protein stabilization. Redox Biol..

[45] Caliò A., Brunelli M., Segala D., Zamboni G., Bonetti F., Pea M., Martignoni G. (2021). Angiomyolipoma of the kidney: From simple hamartoma to complex tumour. Pathology.

[46] Martignoni G., Pea M., Zampini C., Brunelli M., Segala D., Zamboni G., Bonetti F. (2015). PEComas of the kidney and of the genitourinary tract. Semin. Diagn. Pathol..

[47] Giannikou K., Malinowska I.A., Pugh T.J., Yan R., Tseng Y.-Y., Oh C., Kim J., Tyburczy M.E., Chekaluk Y., Liu Y. (2016). Whole Exome Sequencing Identifies TSC1/TSC2 Biallelic Loss as the Primary and Sufficient Driver Event for Renal Angiomyolipoma Development. PLoS Genet..

[48] Motyckova G., Weilbaecher K.N., Horstmann M., Rieman D.J., Fisher D.Z. (2001). Linking osteopetrosis and pycnodysostosis: Regulation of cathepsin K expression by the microphthalmia transcription factor family. Proc. Natl. Acad. Sci. USA.

[49] Martignoni G., Bonetti F., Chilosi M., Brunelli M., Segala D., Amin M.B., Argani P., Eble J.N., Gobbo S., Pea M. (2011). Cathepsin K expression in the spectrum of perivascular epithelioid cell (PEC) lesions of the kidney. Mod. Pathol..

[50] Argani P., Aulmann S., Illei P.B., Netto G.J., Ro J., Cho H.-Y., Dogan S., Ladanyi M., Martignoni G., Goldblum J.R. (2010). A Distinctive Subset of PEComas Harbors TFE3 Gene Fusions. Am. J. Surg. Pathol..

[51] Martignoni G., Bonetti F., Pea M., Tardanico R., Brunelli M., Eble J.N. (2002). Renal Disease in Adults With TSC2/PKD1 Contiguous Gene Syndrome. Am. J. Surg. Pathol..

[52] Caliò A., Brunelli M., Gobbo S., Pedron S., Segala D., Argani P., Martignoni G. (2021). Stimulator of interferon genes (STING) immunohistochemical expression in the spectrum of perivascular epithelioid cell (PEC) lesions of the kidney. Pathology.

[53] Siadat F., Trpkov K. (2020). ESC, ALK, HOT and LOT: Three Letter Acronyms of Emerging Renal Entities Knocking on the Door of the WHO Classification. Cancers.

[54] Trpkov K., Hes O., Bonert M., Lopez J.I., Bonsib S.M., Nesi G., Comperat E., Sibony M., Berney D.M., Martinek P. (2016). Eosinophilic, solid, and cystic renal cell carcinoma: Clinicopathologic study of 16 unique, sporadic neoplasms occurring in women. Am. J. Surg. Pathol..

[55] Parilla M., Kadri S., Patil S.A., Ritterhouse L., Segal J., Henriksen K.J., Antic T. (2018). Are Sporadic Eosinophilic Solid and Cystic Renal Cell Carcinomas Characterized by Somatic Tuberous Sclerosis Gene Mutations?. Am. J. Surg. Pathol..

[56] Mehra R., Vats P., Cao X., Su F., Lee N.D., Lonigro R., Premkumar K., Trpkov K., McKenney J.K., Dhanasekaran S.M. (2018). Somatic Bi-allelic Loss of TSC Genes in Eosinophilic Solid and Cystic Renal Cell Carcinoma. Eur. Urol..

[57] Munari E., Settanni G., Caliò A., Segala D., Lonardi S., Sandrini S., Vacca P., Tumino N., Marconi M., Brunelli M. (2021). TSC Loss is a clonal event in Eosinophilic Solid and Cystic (ESC) Renal Cell Carcinoma (RCC): A multiregional tumor sampling study. Mod. Pathol..

[58] Palsgrove D.N., Li Y., Lin M.T., Pallavajjalla A., Gocke C., De Marzo A.M., Matoso A., Netto G.J., Epstein J.I., Argani P. (2018). Eosinophilic Solid and Cystic (ESC) renal cell carcinomas harbor TSC mutations: Molecular Analysis supports an expanding clinicopathologic spectrum. Am. J. Surg. Pathol..

[59] Li Y., Reuter V.E., Matoso A., Netto G.J., Epstein J.I., Argani P. (2018). Re-evaluation of 33 ‘unclassified’ eosinophilic renal cell carcinomas in young patients. Histopathology.

[60] Chen Y.-B., Mirsadraei L., Jayakumaran G., Al-Ahmadie H.A., Fine S.W., Gopalan A., Sirintrapun S.J., Tickoo S.K., Reuter V.E. (2019). Somatic Mutations of TSC2 or MTOR Characterize a Morphologically Distinct Subset of Sporadic Renal Cell Carcinoma With Eosinophilic and Vacuolated Cytoplasm. Am. J. Surg. Pathol..

[61] He H., Trpkov K., Martinek P., Isikci O.T., Maggi-Galuzzi C., Alaghehbandan R., Gill A.J., Tretiakova M., Lopez J.I., Williamson S.R. (2018). “High-grade oncocytic renal tumor”: Morphologic, immunohistochemical, and molecular genetic study of 14 cases. Virchows Arch..

[62] Trpkov K., Williamson S.R., Gill A.J., Adeniran A.J., Agaimy A., Alaghehbandan R., Amin M.B., Argani P., Chen Y.-B., Cheng L. (2021). Novel, emerging and provisional renal entities: The Genitourinary Pathology Society (GUPS) update on renal neoplasia. Mod. Pathol..

[63] Trpkov K., Bonert M., Gao Y., Kapoor A., He H., Yilmaz A., Gill A.J., Williamson S.R., Comperat E., Tretiakova M. (2019). High-grade oncocytic tumour (HOT) of kidney in a patient with tuberous sclerosis complex. Histopathology.

[64] Moch H., Ohashi R. (2021). Chromophobe renal cell carcinoma: Current and controversial issues. Pathology.

[65] Ross H., Martignoni G., Argani P. (2012). Renal Cell Carcinoma With Clear Cell and Papillary Features. Arch. Pathol. Lab. Med..

[66] Caliò A., Brunelli M., Segala D., Pedron S., Remo A., Ammendola S., Munari E., Pierconti F., Mosca A., Bollito E. (2020). Comprehensive analysis of 34 MiT family translocation renal cell carcinomas and review of the literature: Investigating prognostic markers and therapy targets. Pathology.

[67] Caliò A., Segala D., Munari E., Brunelli M., Martignoni G. (2019). MiT Family Translocation Renal Cell Carcinoma: From the Early Descriptions to the Current Knowledge. Cancers.

[68] Caliò A., Brunelli M., Segala D., Pedron S., Tardanico R., Remo A., Gobbo S., Meneghelli E., Doglioni C., Hes O. (2018). t(6;11) renal cell carcinoma: A study of seven cases including two with aggressive behavior, and utility of CD68 (PG-M1) in the differential diagnosis with pure epithelioid PEComa/epithelioid angiomyolipoma. Mod. Pathol..

[69] Iakymenko O.A., Delma K.S., Jorda M., Kryvenko O.N. (2021). Cathepsin K (Clone EPR19992) Demonstrates Uniformly Positive Immunoreactivity in Renal Oncocytoma, Chromophobe Renal Cell Carcinoma, and Distal Tubules. Int. J. Surg. Pathol..

[70] Caron A., Richard D., Laplante M. (2015). The Roles of mTOR Complexes in Lipid Metabolism. Annu. Rev. Nutr..

[71] Kim L.C., Cook R.S., Chen J. (2017). mTORC1 and mTORC2 in cancer and the tumor microenvironment. Oncogene.

[72] Han J., Wei L., Xu W., Lu J., Wang C., Bao Y., Jia W. (2015). CTSK inhibitor exert its anti-obesity effects through regulating adipocyte differentiation in high-fat diet induced obese mice. Endocr. J..

